# Association of subcortical structural shapes with fatigue in neuromyelitis optica spectrum disorder

**DOI:** 10.1038/s41598-022-05531-1

**Published:** 2022-01-28

**Authors:** Jin Myoung Seok, Wanzee Cho, Doo-Hwan Son, Jong Hwa Shin, Eun Bin Cho, Sung Tae Kim, Byoung Joon Kim, Joon-Kyung Seong, Ju-Hong Min

**Affiliations:** 1 Department of Neurology, Soonchunhyang University Hospital Cheonan, Soonchunhyang University College of Medicine, Cheonan, South Korea; 2 Department of Artificial Intelligence, Korea University, Seoul, South Korea; 3 School of Biomedical Engineering, Korea University, Seoul, South Korea; 4 Department of Neurology, Samsung Medical Center, Sungkyunkwan University School of Medicine, 81 Irwon-ro, Gangnam-gu, Seoul, 06351 South Korea; 5 Neuroscience Center, Samsung Medical Center, Seoul, South Korea; 6 Department of Neurology, Gyeongsang Institute of Health Science, Gyeongsang National University School of Medicine, Jinju, South Korea; 7 Department of Neurology, Gyeongsang National University Changwon Hospital, Changwon, South Korea; 8 Department of Radiology, Samsung Medical Center, Sungkyunkwan University School of Medicine, Seoul, South Korea; 9 Interdisciplinary Program in Precision Public Health, Korea University, Seoul, South Korea; 10 Department of Health Sciences and Technology, Samsung Advanced Institute for Health Sciences and Technology (SAIHST), Sungkyunkwan University, Seoul, 135-710 South Korea

**Keywords:** Fatigue, Demyelinating diseases, Brain imaging

## Abstract

Although fatigue is a major symptom in patients with neuromyelitis optica spectrum disorder (NMOSD), the underlying mechanism remains unclear. We explored the relationship between subcortical structures and fatigue severity to identify neural substrates of fatigue in NMOSD. Clinical characteristics with brain magnetic resonance imaging were evaluated in forty patients with NMOSD. Fatigue was assessed using the Functional Assessment of Chronic Illness Therapy-Fatigue (FACIT-fatigue) questionnaire (a higher score indicates less fatigue). We assessed the correlation between subcortical structures and fatigue severity using surface-based shape analysis. Most of the enrolled patients showed fatigue (72.5%; mean FACIT-fatigue score, 34.8 ± 10.8). The FACIT-fatigue score was negatively correlated with Expanded Disability Status Scale and Beck Depression Inventory scores (*r* = − 0.382, *p* = 0.016; *r* = − 0.578, *p* < 0.001). We observed that the right thalamus was the only extracted region for various threshold experiments. Further, patients with lower FACIT-fatigue scores (more fatigue) had decreased local shape volume in the right thalamus. Fatigue is common in patients with NMOSD, and atrophy in the right thalamus is strongly correlated with fatigue severity. The local shape volume of the right thalamus might serve as a biomarker of fatigue in NMOSD.

## Introduction

Fatigue is a major burden in patients with neuroimmunological diseases^1^. In multiple sclerosis (MS), fatigue is the most frequent and disabling symptom reported by patients^1–3^. Neuromyelitis optica spectrum disorder (NMOSD), a severe central nervous system (CNS) autoimmune disorder, is characterised by the presence of anti-aquaporin-4 (anti-AQP4) antibodies and chronic fatigue. Previous studies showed that the incidence of fatigue is 58–77% in patients with NMOSD^4–7^. However, the mechanisms underlying fatigue in patients with NMOSD are poorly understood. Studies also suggest that sleep quality, pain, and depression are the possible factors that are correlated with fatigue severity in NMOSD^4,6,7^. Considering the pathophysiological mechanisms of fatigue, these findings showed only clinical contributors of secondary fatigue in NMOSD^1^.

As a primary fatigue of the disease, the pathologic basis or magnetic resonance imaging (MRI) substrates of fatigue in NMOSD remain unknown. In MS, fatigue caused by the disease itself is a result of brain damage due to inflammation, demyelination, or axonal loss in the CNS, which causes disruption of the brain network or connections^1,8,9^. Several neuroimaging studies have suggested that white matter lesions, cortical or subcortical atrophy, or brain network disruption are correlated with fatigue in patients with MS^10–14^. In NMOSD, the MRI substrate of fatigue has not been investigated previously, although characteristic brain lesions with white matter diffusion abnormalities and brain atrophy have been studied^15–17^.

Herein, we explored the brain subcortical structures and investigated their relationship with fatigue severity in patients with NMOSD using surface-based shape analysis.

## Results

### Demographic and clinical features

Forty patients with NMOSD (32 female, 80.0%; mean age, 47.8 ± 12.4 years) were enrolled in this study. Most of the patients (34, 85.0%) were seropositive for anti-AQP4 antibodies. The median disease duration was 2.9 years (inter-quartile range (IQR), 1.1–7.1 years), and the mean Expanded Disability Status Scale (EDSS) score was 2.8 ± 2.3. Twenty-two patients (55.0%) had either brain or brainstem syndrome during the disease course, which was diagnosed before the MRI scan. Mycophenolate mofetil was the most commonly used drug for preventive treatment (18 patients, 45.0%). Details of the clinical involvement and preventive treatment are presented in Table 1.

**Table 1:** Clinical characteristics of enrolled patients with neuromyelitis optica spectrum disorder.

	Enrolled patients with NMOSD N = 40
Age, years (SD)	47.8 (12.4)
Female, n (%)	32 (80.0)
AQP4 antibody, n (%)	34 (85.0)
Disease duration, years (IQR)	2.9 (1.1–7.1)
EDSS (SD)	2.8 (2.3)
Number of relapses (IQR)	2 (1 − 4)
**Type of relapse experience**^**†**^	
Optic neuritis, n (%)	24 (60.0)
Transverse myelitis, n (%)	23 (57.5)
Area of postrema syndrome, n (%)	4 (10.0)
Brainstem syndrome, n (%)	16 (40.0)
Cerebral syndrome, n (%)	13 (32.5)
**Preventive treatment**	
Azathioprine, n (%)	12 (30.0)
Mycophenolate mofetil, n (%)	18 (45.0)
Rituximab, n (%)	4 (10.0)
Oral prednisolone, n (%)	1 (2.5)
No treatment, n (%)	4 (10.0)
Other^‡^, n (%)	1 (2.5)
FACIT-fatigue score, (SD)	34.8 (10.8)
Beck Depression Inventory score, (SD)	14.1 (8.5)

*NMOSD* neuromyelitis optica spectrum disorder, *SD* standard deviation, *IQR* interquartile range, *AQP4* Aquaporin-4, *FACIT-fatigue* Functional Assessment of Chronic Illness Therapy-fatigue.

^†^Onset attacks are included.

^‡^Eculizumab (n = 1) was used for preventive treatment.

### Self-reported questionnaires for fatigue and depression

**Figure 1:**
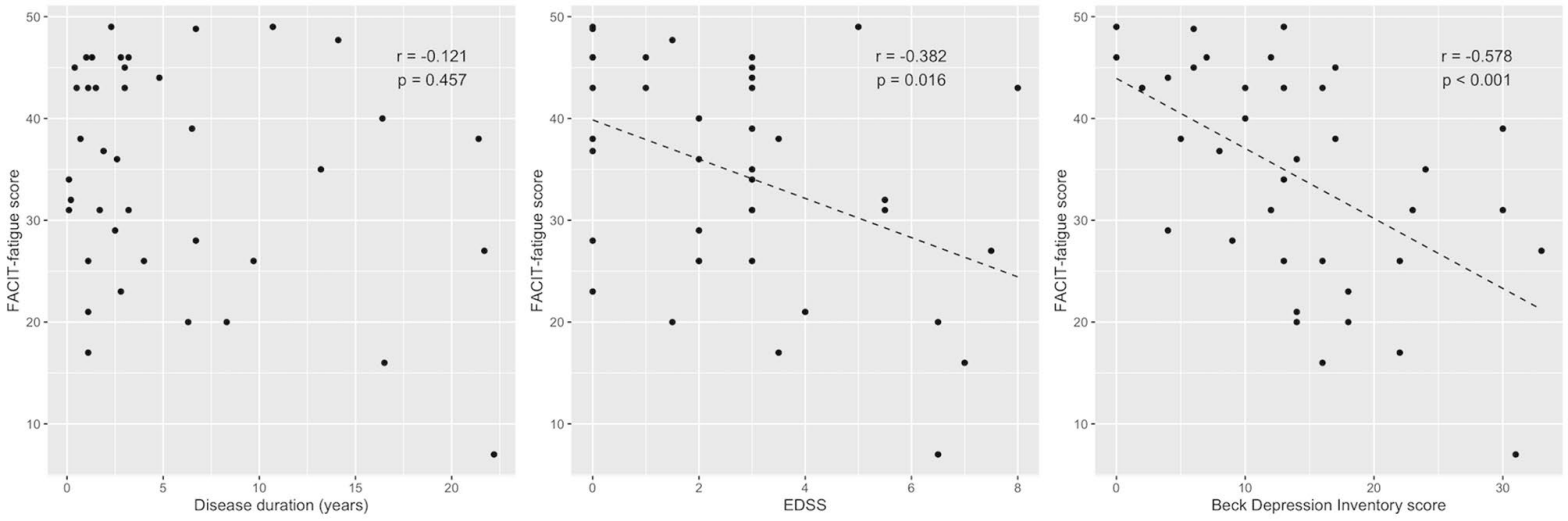
Scatter plots depicting correlation among disease duration, EDSS, BDI, and FACIT-fatigue. The FACIT-fatigue score was negatively correlated with EDSS and BDI scores; however, no correlation was observed between the FACIT-fatigue score and disease duration.

All patients completed the Functional Assessment of Chronic Illness Therapy-Fatigue scale (FACIT-fatigue) and Beck Depression Inventory (BDI) questionnaires. The mean FACIT-fatigue score was 34.8 ± 10.8. A previous study conducted in the United States with 1075 individuals showed that the median FACIT-fatigue score in general population was 43^18^. Based on this cut-off, most of our patients with NMOSD (72.5%) showed fatigue. The mean BDI score was 14.1 ± 8.5, and 20.0% (n = 8) of the enrolled patients had moderate or severe depression. The FACIT-fatigue score was negatively correlated with EDSS and BDI scores (*r* = − 0.382, *p* = 0.016; *r* = − 0.578, *p* < 0.001, respectively); however, no correlation was observed between FACIT-fatigue score and disease duration (*r* = − 0.121, *p* = 0.457) (Fig. 1).

### Shape analysis

**Figure 2:**
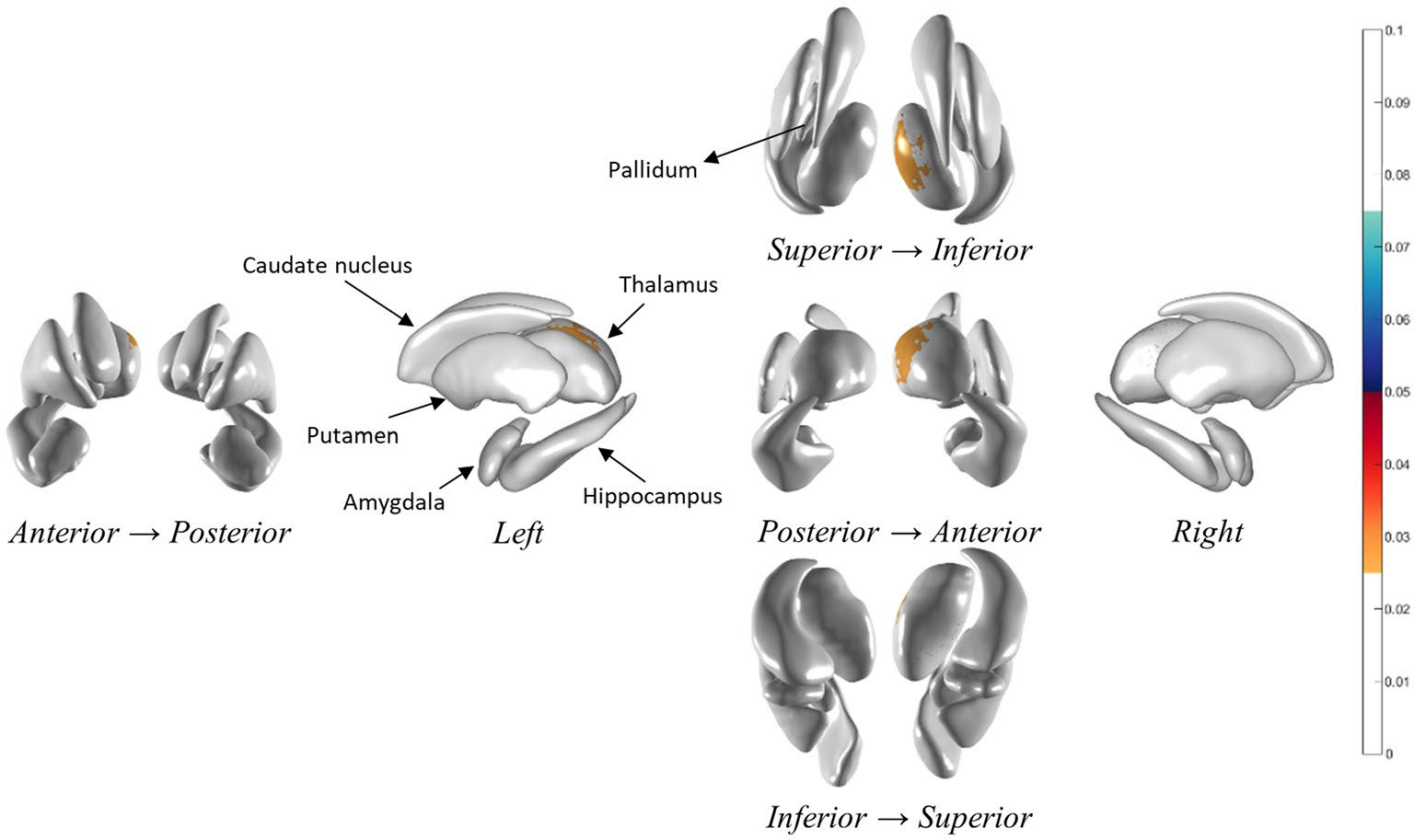
Significant regions related with FACIT-fatigue score at a threshold of 0.35. The area of decreased local shape volume correlated with FACIT-fatigue score involved the right thalamus in patients with NMOSD.

To extract the domains in which fatigue scores were correlated, we performed a correlation analysis between the subcortical local shape volume and FACIT-fatigue scores in patients with NMOSD. The covariates for the partial correlation coefficients were age, sex, intracranial volume, and BDI score. We observed that the right thalamus is the only extracted region for various threshold experiments; significant subcortical regions and *p*-values for each threshold experiment are presented in Supplementary Table 1. Significant results were extracted only with the positive thresholds, suggesting that patients with a lower FACIT-fatigue score had decreased local shape volume (Fig. 2).

## Discussion

In this study, we observed that 72.5% of patients with NMOSD had fatigue, measured using the FACIT-fatigue score. Furthermore, fatigue severity correlated with the local shape volume of the right thalamus after adjusting for age, sex, and intracranial volume together with the depression score.

Brain involvement is frequently reported in NMOSD, and MRI findings of characteristic brain lesions in NMOSD include peri-ependymal lesions surrounding the ventricles, hemispheric large lesions, corticospinal tract lesions, and ‘cloud-like’ enhancing lesions^15^. In addition, recent studies have demonstrated the involvement of cortical grey and deep grey matter in NMOSD. A previous study focused on the deep grey matter observed selective atrophy of the thalamus in patients with NMOSD^19^. Another study showed that in NMOSD, significant grey matter volume reduction affects the frontal/temporal cortices and right thalamus, and is correlated with cognitive dysfunction^20^. Our study showed that changes in thalamic volume correlates with fatigue severity. To the best of our knowledge, this study is the first to identify the MRI substrate of fatigue in patients with NMOSD, and demonstrated the importance of the thalamus in the pathophysiology of fatigue in NMOSD.

The thalamus is involved in regulating several brain functions, including motor, sensory, and higher cortical functions, and acts as a gateway between cortical and subcortical areas^13,14^. The relationship between the thalamus and fatigue has been studied in patients with MS. Several neuropathological and neuroimaging studies have reported the involvement of thalamus in MS^13,21,22^, and dysfunction of the cortico-subcortical pathway with thalamus atrophy is considered as a major substrate of fatigue in patients with MS^2,10,13^. Besides the morphological alterations, some authors suggested that the microstructural changes of the thalamus assessed by diffusion tensor imaging (DTI) could also be related to fatigue in the early stages of MS^23^. And the others showed DTI parameters from cortex, especially right temporal cortex, correlated with thalamic atrophy and fatigue severity^24^. Multimodal MRI studies of fatigue in NMOSD are needed to find more precise imaging biomarkers for fatigue.

Interestingly, we observed a significant correlation between fatigue severity and local shape volume loss only in the right thalamus. A previous study reported whole thalamic grey matter atrophy in patients with NMOSD^19^, and right or left deep grey matter volumes were not measured separately. Another study on grey matter volume and cognition showed that right thalamus volume loss is correlated with reduced cognitive function in patients with NMOSD^20^. Studies using functional MRI suggested that the right prefrontal cortex is related to mental fatigue, which might be because right lateralised activity is associated with sustained attention^25,26^. And MRI studies of neurodegenerative diseases reported that the laterality of thalamic atrophy might be related to the dominant presence of the right-handedness and the tested cognitive functions. Therefore, the handedness might be related to fatigue and thalamic volume. However, the handedness was not considered in our study. Further studies on the laterality of the thalamus with respect to fatigue in patients NMOSD are needed for a detailed understanding.

In this study, subcortical shape alterations were analysed in association with several clinical scores based on subcortical mesh surfaces. Using surface-based measurements for local shape atrophy, statistical analysis becomes more sensitive compared to volume-based morphometry analysis. Furthermore, the exploitation of cluster-based statistics provides statistical correction for multiple comparisons, and is known to be more sensitive than other correction methods, such as false discovery rate and Bonferroni correction.

Depression is one of the most common comorbidities of neurological diseases, and the severity of depression is correlated with fatigue^4,6,7^. In our study, although 20.0% of the patients had moderate or severe depression, they have not received any treatment for depression. A previous study also showed that depression in patients with NMOSD is associated with fatigue and is insufficiently treated^27^. Further studies are required to address whether antidepressant treatment could improve fatigue, and will help understand the relationship between depression and fatigue in NMOSD.

Our study has some limitations. First, this was a cross-sectional study performed at a single centre with a relatively small sample size, which may limit the generalisation of our findings to all patients with NMOSD. To overcome this limitation, we performed a comprehensive clinical and imaging analysis for patients with NMOSD, and all patients underwent anti-AQP4 antibody and anti-myelin oligodendrocyte glycoprotein (anti-MOG) antibody tests at the time of enrolment, which could help distinguish between seronegative NMOSD and MOG-associated disease. Second, we did not include a healthy control group. The proposed statistical method, however, extracts statistically significant subcortical regions associated with several clinical scores, such as fatigue, using cluster-based statistics even without control data. Finally, other factors such as pain or sleep problems that cause secondary fatigue in NMOSD were not considered in this study.

In conclusion, fatigue is common in patients with NMOSD, and atrophy in the right thalamus is strongly associated with fatigue severity. Moreover, local shape volume of the right thalamus might serve as a biomarker of fatigue in patients with NMOSD. Further longitudinal studies will help to establish the role of MRI analysis as a predictor of fatigue in NMOSD.

## Methods

### Patients

We prospectively studied patients with NMOSD who visited the outpatient clinic of neurology at the Samsung Medical Center (Seoul, Korea) between May 2016 and May 2020. Patients were enrolled if they met the international consensus diagnostic criteria for NMOSD^28^ and were in remission phase for at least 6 months. All enrolled patients completed a self-report questionnaire for the assessment of fatigue and depression, and underwent brain MRI at the time of questionnaire assessment. Standardised^26^ T2-weighted, three-dimensional T1-weighted turbo field echo, and three-dimensional fluid-attenuated inversion recovery images were acquired using a 3.0-T MRI scanner (Philips 3.0 T Achieva, Philips Healthcare, Andover, MA, USA) as previously described^29^. Patients were excluded from the study if (a) anti-AQP4 and anti-MOG antibodies were not assessed, (b) they refused to participate in the study, and (c) they had medical disorders including major depressive disorders, narcolepsy, or chronic infectious diseases like tuberculosis which could be related to fatigue, (d) they were taking medications like antidepressants or modafinil that could alter the status of fatigue or depression. We assessed the clinical characteristics of the enrolled patients, including age, sex, disease duration, clinical relapses, disability, and current treatments such as immunosuppressants or oral prednisolone. Disability was assessed using the EDSS.

The study was approved by the local ethics committees of the Samsung Medical Center; all participants provided written informed consent prior to commencement of the study, and all methods were performed in accordance with the relevant guidelines and regulations.

### Instruments

The Korean version of the FACIT-fatigue was used to assess fatigue. FACIT-fatigue is a 13-item self-report questionnaire with final scores ranging from 0 to 52; a higher FACIT-fatigue score indicates less fatigue^30^. The FACIT-fatigue has been validated and used in patients with autoimmune disorders, including NMOSD^6,31–33^. We evaluated the severity of depression using the BDI, which includes 21 items. BDI scores range from 0 to 63; higher scores indicate more severe depression.

### Image processing and analysis

**Figure 3:**
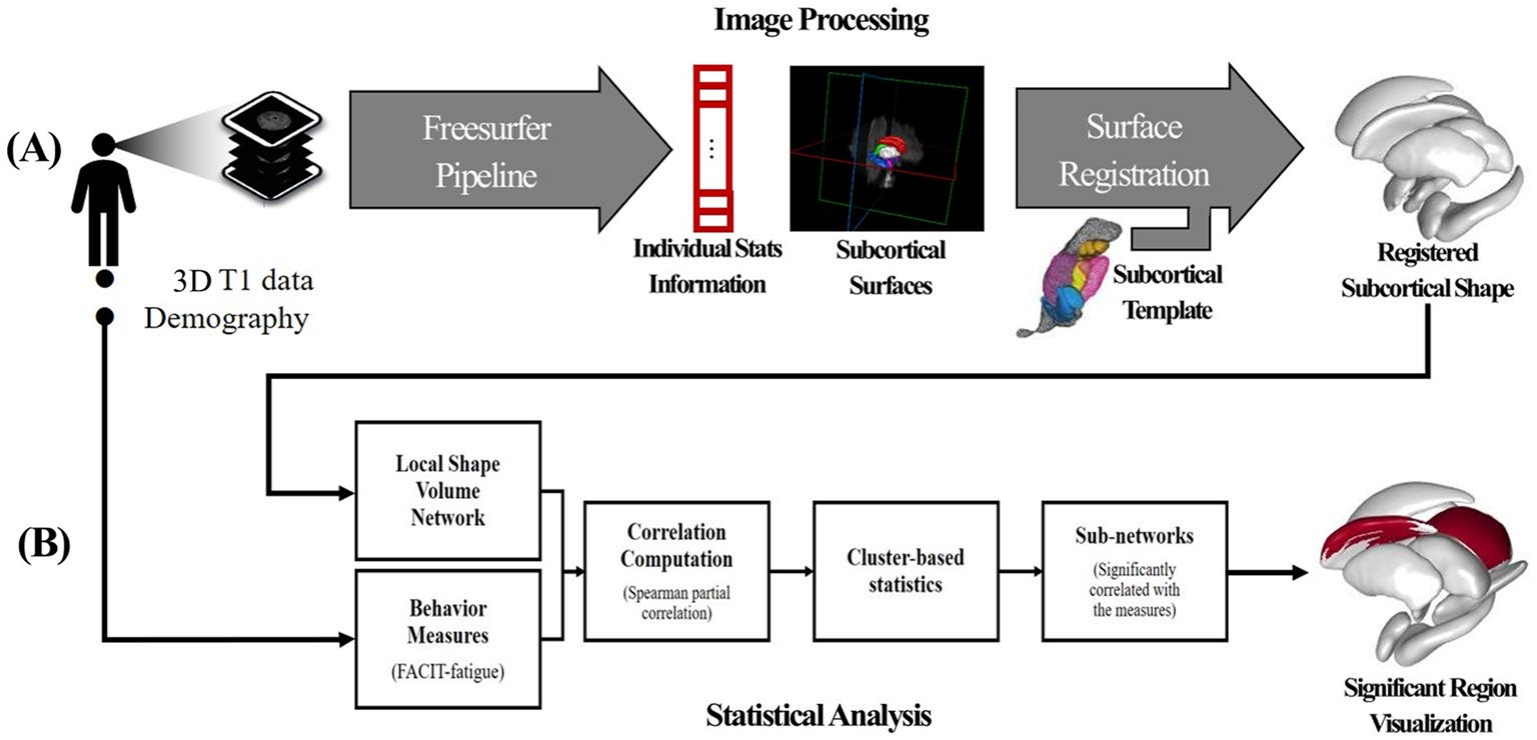
Overview of the proposed method. (**A**) Subcortical shape analysis, (**B**) statistical analysis using cluster-based statistics and visualising statistically significant subnetworks for each subcortical region.

MR images were processed to extract subcortical shapes for each individual, which were then registered to a template subcortical surface. After local shape volume extraction, together with demographic information, we performed a statistical analysis of the subcortical regions with partial correlation computation and extracted significant sub-clusters via multiple comparison correction using cluster-based statistics (Fig. 3).

The T1-weighted MR images were preprocessed to extract the subcortical surfaces, which were then used to calculate the local shape volume for each vertex. This process consists of four steps: volume parcellation, surface extraction, surface registration, and local shape volume calculation. In the first step, we parcelled the anatomy of human subcortical structures from a T1-weighted image for each patient using the Freesurfer software package (version 6.0.0; Athinoula A. Martinos Center at the Massachusetts General Hospital, Harvard Medical School; http://www.surfer.nmr.mgh.harvard.edu/). The parcelled images were transformed into a native anatomical space for surface-mesh extraction. In the second step, surface meshes were extracted for each patient by deforming the template surface model. Specifically, subcortical shape atlas models^34^ were used as a template surface, and the Laplacian-based surface deformation method^35,36^ was used to extract the subcortical surface of each patient. In the third step, the surface registration method developed by Cho et al.^37^ was used to establish the vertex correspondence of the subcortical surface meshes across samples. In the final step, the local shape volume of each vertex was measured using the method proposed by Shapira et al.^38^. By definition, this process measures the local shape volume at each vertex. Each vertex of the registered surface then has a local shape volume score, which encodes the information on the local cone-shaped volume towards the normal vector of the vertex. Therefore, the local shape volume can be used to analyse the surface-based atrophy of subcortical structures. Each subcortical deep nucleus is composed of 2562 vertices. We analysed the corresponding vectors together with the FACIT-fatigue score, sex, and BDI score.

### Statistical analysis

Clinical characteristics of the enrolled patients are presented with appropriate summary statistics. Continuous data are shown as mean with standard deviation or median with the IQR. Categorical variables are presented as absolute and relative frequencies. Spearman’s correlation was used to evaluate the association between the FACIT-fatigue score and other variables.

Statistical analysis of the MRI data was performed according to a previously reported protocol^39^. To investigate the correlation between subcortical local shape volume and FACIT-fatigue scores, we used nonparametric Spearman partial correlation coefficients. The partial correlation coefficient is useful for reducing the effects of other confounding factors in the analysis. As changes in subcortical volume can be associated with normal ageing, sex differences, intracranial volume differences, and the level of depression, we used age, sex, intracranial volume, and BDI score as covariates. We calculated the partial correlation coefficients for every vertex of one subcortical mesh. We provided information on the adjacency matrix (2562 × 2562) to specify which vertices are connected to the other. We set the initial threshold of the correlation coefficients from − 0.4 to 0.4. With each initial threshold and adjacency matrix, we obtained subnetworks that are clustering-connected supra-threshold vertices. A multiple comparison correction using the cluster-based statistical method was performed. Representative statistics were calculated by counting the nodes of the largest connected subnetwork of each permutation (maximal cluster extent). A null permutation distribution was formed with the maximal cluster extent. We estimated the significance level over the null distribution by computing the proportion of entries with maximal cluster extent, entries that are larger than the size of each identified subnetwork, the maximal cluster extent of the original ordering of fatigue scores, and the number of entries. We performed the permutation test with 5000 permutations to identify connections that are correlated with the fatigue score. All statistical analyses were performed using either MATLAB (R2017a) or R software version 3.6.3. Statistical significance was defined as a two-tailed *p*-value < 0.05. All significant clusters in each subcortical region were visualised.

## Supplementary Information

Supplementary Table 1.

## References

[1] Penner IK, Paul F (2017). Fatigue as a symptom or comorbidity of neurological diseases. Nat. Rev. Neurol..

[2] Induruwa I, Constantinescu CS, Gran B (2012). Fatigue in multiple sclerosis—A brief review. J. Neurol. Sci..

[3] Krupp L (2006). Fatigue is intrinsic to multiple sclerosis (MS) and is the most commonly reported symptom of the disease. Mult. Scler..

[4] Akaishi T, Nakashima I, Misu T, Fujihara K, Aoki M (2015). Depressive state and chronic fatigue in multiple sclerosis and neuromyelitis optica. J. Neuroimmunol..

[5] Pan J (2015). Hypoxemia, sleep disturbances, and depression correlated with fatigue in neuromyelitis optica spectrum disorder. CNS Neurosci. Ther..

[6] Seok JM (2017). Fatigue in patients with neuromyelitis optica spectrum disorder and its impact on quality of life. PLoS One.

[7] Yeo T (2020). Factors associated with fatigue in CNS inflammatory diseases with AQP4 and MOG antibodies. Ann. Clin. Transl. Neurol..

[8] Filippi M (2002). Functional magnetic resonance imaging correlates of fatigue in multiple sclerosis. Neuroimage.

[9] Kos D, Kerckhofs E, Nagels G, D’Hooghe MB, Ilsbroukx S (2008). Origin of fatigue in multiple sclerosis: Review of the literature. Neurorehabil. Neural Repair.

[10] Sepulcre J (2009). Fatigue in multiple sclerosis is associated with the disruption of frontal and parietal pathways. Mult. Scler..

[11] Pellicano C (2010). Relationship of cortical atrophy to fatigue in patients with multiple sclerosis. Arch. Neurol..

[12] Bisecco A (2016). Fatigue in multiple sclerosis: The contribution of occult white matter damage. Mult. Scler..

[13] Minagar A (2013). The thalamus and multiple sclerosis: Modern views on pathologic, imaging, and clinical aspects. Neurology.

[14] Capone F, Collorone S, Cortese R, Di Lazzaro V, Moccia M (2020). Fatigue in multiple sclerosis: The role of thalamus. Mult. Scler..

[15] Kim HJ (2015). MRI characteristics of neuromyelitis optica spectrum disorder: An international update. Neurology.

[16] Kim SH (2016). Widespread cortical thinning in patients with neuromyelitis optica spectrum disorder. Eur. J. Neurol..

[17] Jeong IH (2017). Normal-appearing white matter demyelination in neuromyelitis optica spectrum disorder. Eur. J. Neurol..

[18] Cella D, Lai JS, Stone A (2011). Self-reported fatigue: One dimension or more? Lessons from the Functional Assessment of Chronic Illness Therapy-Fatigue (FACIT-F) questionnaire. Support Care Cancer.

[19] Hyun JW (2017). Deep gray matter atrophy in neuromyelitis optica spectrum disorder and multiple sclerosis. Eur. J. Neurol..

[20] Wang Q (2015). Gray matter volume reduction is associated with cognitive impairment in neuromyelitis optica. AJNR Am. J. Neuroradiol..

[21] Popescu BF, Lucchinetti CF (2012). Meningeal and cortical grey matter pathology in multiple sclerosis. BMC Neurol..

[22] Houtchens MK (2007). Thalamic atrophy and cognition in multiple sclerosis. Neurology.

[23] Wilting J (2016). Structural correlates for fatigue in early relapsing remitting multiple sclerosis. Eur. Radiol..

[24] Bernitsas E (2017). Structural and neuronal integrity measures of fatigue severity in multiple sclerosis. Brain Sci..

[25] Cabeza R, Nyberg L (2000). Imaging cognition II: An empirical review of 275 PET and fMRI studies. J. Cogn. Neurosci..

[26] Cook DB, O'Connor PJ, Lange G, Steffener J (2007). Functional neuroimaging correlates of mental fatigue induced by cognition among chronic fatigue syndrome patients and controls. Neuroimage.

[27] Chavarro VS (2016). Insufficient treatment of severe depression in neuromyelitis optica spectrum disorder. Neurol. Neuroimmunol. Neuroinflamm..

[28] Wingerchuk DM (2015). International consensus diagnostic criteria for neuromyelitis optica spectrum disorders. Neurology.

[29] Cho EB (2018). White matter network disruption and cognitive dysfunction in neuromyelitis optica spectrum disorder. Front. Neurol..

[30] Yellen SB, Cella DF, Webster K, Blendowski C, Kaplan E (1997). Measuring fatigue and other anemia-related symptoms with the Functional Assessment of Cancer Therapy (FACT) measurement system. J. Pain Symptom Manag..

[31] Haldorsen K, Bjelland I, Bolstad AI, Jonsson R, Brun JG (2011). A five-year prospective study of fatigue in primary Sjogren's syndrome. Arthritis Res. Ther..

[32] Lai JS, Beaumont JL, Ogale S, Brunetta P, Cella D (2011). Validation of the functional assessment of chronic illness therapy-fatigue scale in patients with moderately to severely active systemic lupus erythematosus, participating in a clinical trial. J. Rheumatol..

[33] Traboulsee A (2020). Safety and efficacy of satralizumab monotherapy in neuromyelitis optica spectrum disorder: A randomised, double-blind, multicentre, placebo-controlled phase 3 trial. Lancet Neurol..

[34] Qiu A, Fennema-Notestine C, Dale AM, Miller MI, Alzheimer's Disease Neuroimaging Initiative (2009). Regional shape abnormalities in mild cognitive impairment and Alzheimer's disease. Neuroimage.

[35] Kim J-I, Park J (2012). Organ shape modeling based on the Laplacian deformation framework for surface-based morphometry studies. J. Comput. Sci. Eng..

[36] Sorkine O (2006). Differential representations for mesh processing. Comput. Graph Forum.

[37] Cho Y, Seong JK, Jeong Y, Shin SY, Neuroimaging AD (2012). Individual subject classification for Alzheimer's disease based on incremental learning using a spatial frequency representation of cortical thickness data. Neuroimage.

[38] Shapira L, Shamir A, Cohen-Or D (2008). Consistent mesh partitioning and skeletonisation using the shape diameter function. Visual Comput..

[39] Han CE, Yoo SW, Seo SW, Na DL, Seong JK (2013). Cluster-based statistics for brain connectivity in correlation with behavioral measures. PLoS One.

