# Supplementary material for: Association of subcortical structural shapes with fatigue in neuromyelitis optica spectrum disorder
Source: Sci Rep. 2022 Jan 28;12:1579. doi: 10.1038/s41598-022-05531-1 (PMC8799731; doi:10.1038/s41598-022-05531-1)
Supplement: Supplementary file 1 — Supplementary Table 1. [file 41598_2022_5531_MOESM1_ESM.docx]

Supplementary Table 1. Significant subcortical regions correlated with the FACIT-fatigue score^*^

| Significant cluster | *r* (threshold) | *p*-value |
| --- | --- | --- |
| Right thalamus | 0.2 | 0.0062 |
| Right thalamus | 0.25 | 0.0060 |
| Right thalamus | 0.3 | 0.0058 |
| Right thalamus | 0.35 | 0.0056 |
| Right thalamus | 0.4 | 0.0056 |

^*^Covariates for partial correlation were the age, intracranial volume, gender and BDI score.
